# The Characteristic Properties of Magnetostriction and Magneto-Volume Effects of Ni_2_MnGa-Type Ferromagnetic Heusler Alloys

**DOI:** 10.3390/ma12223655

**Published:** 2019-11-06

**Authors:** Takuo Sakon, Yuushi Yamasaki, Hiroto Kodama, Takeshi Kanomata, Hiroyuki Nojiri, Yoshiya Adachi

**Affiliations:** 1 Department of Mechanical and Systems Engineering, Faculty of Science and Technology, Ryukoku University, Otsu, Shiga 520-2194, Japan; t160260@mail.ryukoku.ac.jp (Y.Y.); t150246@mail.ryukoku.ac.jp (H.K.); 2 Research Institute for Engineering and Technology, Tohoku Gakuin University, Tagajo, Miyagi 985-8537, Japan; c1924007@mail.tohoku-gakuin.ac.jp; 3 Institute for Materials Research, Tohoku University, Sendai, Miyagi 980-8577, Japan; nojiri@imr.tohoku.ac.jp; 4 Graduate School of Science and Engineering, Yamagata University, Yonezawa, Yamagata 992-8510, Japan; adachy@yz.yamagata-u.ac.jp

**Keywords:** Heusler alloy, ferromagnet, magnetization, magnetostriction, itinerant electron ferromagnetism

## Abstract

In this article, we review the magnetostriction and magneto-volume effects of Ni_2_MnGa-type ferromagnetic Heusler alloys at the martensitic, premartensitic, and austenitic phases. The correlations of forced magnetostriction (*ΔV*/*V*) and magnetization (*M*), using the self-consistent renormalization (SCR) spin fluctuation theory of an itinerant electron ferromagnet proposed by Takahashi, are evaluated for the ferromagnetic Heusler alloys. The magneto-volume effect occurs due to the interaction between the magnetism and volume change of the magnetic crystals. The magnetic field-induced strain (referred to as forced magnetostriction) and the magnetization are measured, and the correlation of magnetostriction and magnetization is evaluated. The forced volume magnetostriction *ΔV*/*V* at the Curie temperature, *T*_C_ is proportional to *M*^4^, and the plots cross the origin point; that is, (*M*^4^, *ΔV*/*V*) = (0, 0). This consequence is in good agreement with the spin fluctuation theory of Takahashi. An experimental study is carried out and the results of the measurement agree with the theory. The value of forced magnetostriction is proportional to the valence electron concentration per atom (*e*/*a*). Therefore, the forced magnetostriction reflects the electronic states of the ferromagnetic alloys. The magnetostriction near the premartensitic transition temperature (*T*_P_) induces lattice softening; however, lattice softening is negligible at *T*_C_. The forced magnetostriction at *T*_C_ occurs due to spin fluctuations of the itinerant electrons. In the martensitic and premartensitic phases, softening of the lattice occurs due to the shallow hollow (potential barrier) of the total energy difference between the L2_1_ cubic and modulated 10M or 14M structures. As a result, magnetostriction is increased by the magnetic field.

## 1. Introduction

Ferromagnetic shape-memory alloys (FSMAs) have been investigated intensively as highly functional materials for use as magnetic actuators, oscillators, magnetic sensors, and magnetic refrigerators. Among FSMAs, Ni_2_MnGa is the most famous alloy [1]. The crystal structure is the Heusler type L2_1_ (Fm $\bar{3}$ m) cubic structure. A ferromagnetic transition occurs at the Curie temperature *T*_C_ ≈ 370 K [2,3]. At the martensitic transition temperature *T*_M_ = 200 K, a martensitic transition occurs as a structural transformation. In the martensitic phase, a lattice modulation takes place. As a result, a superstructure state appears [4,5]. A large strain occurs during martensitic transition. In the martensitic phase, rearrangements of variants can be caused by magnetic fields. This phenomenon has been called “twinning magnetostriction” [1,6]. In Ni_2_MnGa-type single crystals, magnetic field induced strains (MFISs) of 6–10% have been observed near or below room temperature and in the martensitic phase [7]. Predominant magnetostriction has also been observed at the premartensitic (precursor) phase in Ni_2_MnGa. A minimum of the magnetostriction has been found at around the premartensitic temperature, *T*_p_ in a Ni_2_MnGa single crystal [8,9]. The minimum of the elastic modulus was also found around *T*_p_. Matsui et al. [10] investigated magnetostriction in Ni_2_MnGa-type alloys and found a −190 ppm magnetostriction in the premartensitic phase, which is more than 3 times that in the austenitic phase.

Some researchers have investigated the magnetism of Ni_2_MnGa-type Heusler alloys by means of spin fluctuation theories [11,12,13,14,15,16,17,18]. Spin fluctuation theories for the itinerant electron magnetism have been used to evaluate the physics of the itinerant electron system [11,12,19,20,21,22]. According to the self-consistent renormalization (SCR) spin fluctuation theory, referred to as the Moriya theory, the magnetic field *H* is proportional to *M*^3^ [19]. In this theory, the lateral modes of thermally activated spin fluctuations are considered [19,21,22]. Takahashi proposed the spin fluctuation theory of itinerant electron magnetism in compliance with zero-point spin fluctuations [12], in which the amplitude of the total local spin fluctuation, comprised of zero-point and thermal spin fluctuation amplitudes, is preserved. In this theory, the external magnetic field relies on the magnetization at *T*_C_. Takahashi’s theory suggests that the magnetic field *H* is proportional to *M*^5^ at *T*_C_ [12]. This relation has been observed for MnSi [11], Ni [13], CoS_2_ [14], Fe [15], and Fe*_x_*Co_1−*x*_Si [23]. For Ni_2_MnGa, the plot of *M*^4^ has been shown to be proportional to *H*/*M* through the origin point at *T*_C_ [13].

The magneto-volume effect is caused by interaction between the magnetism and the lattice distortion of the magnetic crystals. Takahashi investigated the effects of spin fluctuations on the volume change of magnetic crystals [12]. For magnetostriction, anomalous behavior from the forced volume magnetostriction (strain applied to a magnetic field in the isothermal state) has been observed due to itinerant spin fluctuations near the Curie temperature *T_C_*. The forced volume magnetostriction *ΔV*/*V* is given by the volume differential of free energy.

The relationship of the forced volume magnetostriction *ΔV*/*V* is proportional to *M*^4^ at *T*_C_ [12]. Matsunaga et al. investigated the magnetostriction in a weak itinerant ferromagnet MnSi [24]. They plotted the longitudinal magnetostriction *ΔL*/*L* versus *M*^2^. For *T*_C_ = 30 K, the plot became nonlinear. Takahashi suggested that *ΔL*/*L* is proportional to *M*^4^ through the origin at *T* = 29 K near *T*_C_ [12].

In a previous study, we investigated the magnetization and the magnetostriction of Ni_2+*x*_MnGa_1__−*x*_ (*x* = 0.00, 0.02, and 0.04) to determine whether these relations were preserved when the valence electron concentration per atom, *e*/*a,* changed [16,17,18]. When the value of *x* for Ni_2+*x*_MnGa_1__−*x*_ increased, *e*/*a* increased. The obtained magnetization values for *x* = 0.00 (*e*/*a* = 7.500), *x* = 0.02 (*e*/*a* =7.535), and *x* = 0.04 (*e*/*a* = 7.570) showed that the relation *H* ∝ *M*^5^ can be used at *T*_C_. The plot of magnetostriction versus *M*^4^ was proportional and crossed the origin point. These results are explained by Takahashi’s theory of spin fluctuations. In this study, we measure the magnetization and magnetostriction processes of Ni_2_Mn_1__−*x*_Cr*_x_*Ga for *x* = 0.15 (*e*/*a* = 7.460) and *x* = 0.25 (*e*/*a* = 7.375) in the magnetic field. Moreover, we investigate the relationship between magnetization and magnetostriction at *T*_C_, in accordance with the Takahashi SCR theory.

The forced volume magnetostriction *ΔV*/*V* and the magnetization *M* at *T*_C_ can be described by [12]:

(1) $$\left(\Delta V/V\right)\propto M^{4},$$

where *ΔV*/*V* can be derived by the following equation:

(2) $$\left(\Delta V/V\right)={\left(∆/L\right)}_{//}+2\times {\left(\Delta L/L\right)}_{⊥},$$

where, (*∆L*/*L*)_//_ and ${\left(\Delta L/L\right)}_{⊥}$ are the forced linear magnetostriction parallel and perpendicular to an external magnetic field, respectively [25,26].

In this study, we consider the characteristics of magnetostriction and magneto-volume effects of Ni_2_MnGa-type ferromagnetic Heusler alloys at the martensitic, premartensitic, and austenitic phases, while referring to the electric states. We measure the forced longitudinal magnetostriction (*∆L*/*L*)_//_ and ${\left(\Delta L/L\right)}_{⊥}$, derive the forced volume magnetostriction ΔV/V as shown by Equation (2), and evaluate the correlation between the magnetization and ΔV/V. We also discuss the origin of magnetostriction in the martensitic and premartensitic (precursor) phases, as well as at *T*_C_, using the experimental and theoretical results concerning the band structures.

## 2. Materials and Methods

Polycrystal Ni_2_Mn_1−*x*_Cr*_x_*Ga (*x* = 0.00, 0.15, and 0.25) alloys were synthesized by repeated arc-melting processes of the constituent elements (3N Ni, 4N Mn, 4N Cr, and 6N Ga) in an argon atmosphere. The reaction products were encapsulated in evacuated silica tubes and heated at 1073 K for 3 days and 773 K for 2 more days, then quenched in water. A detailed explanation of the experimental procedure has been given in a previous studies [16,27].

## 3. Results and Discussion

### 3.1. Magnetic Field Dependency of the Magnetization

**Figure 1:**
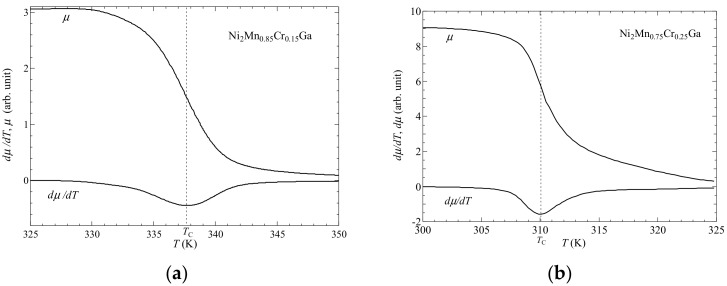
Permeability *μ* and *dμ/dT* for (**a**) *x* = 0.15 and (**b**) *x* = 0.25 near *T*_C_. Data from Ref. [27]. T. Sakon, et al. *Metals* **2017**, *7*, 410. MDPI, doi:10.3390/met7100410.

Figure 1 shows the temperature dependency of the permeability *μ* for (a) *x* = 0.15 and (b) *x* = 0.25, respectively, in a zero external magnetic field. An *L2*_1_-type austenitic phase was observed near *T*_C_. The values of *dμ*/*dT* shown in Figure 1 are the values of the permeability *μ* differentiated with respect to temperature. For *x* = 0.15 and 0.25, the value of *T*_C_ was obtained from the peak of *dμ*/*dT*, which were 338 K and 310 K, respectively. The value of *T*_C_ for *x* = 0.00 (Ni_2_MnGa) has been found to be 375 K, using the same approach [16]. For 0≤x≤0.25, the premartensitic phase was observed before the martensitic transition occurred [27,28,29]. Singh et al. performed X-ray measurements and demonstrated that the crystal structure of Ni_2_MnGa in the premartensitic phase was a 3M-like incommensurate structure [30].

**Figure 2:**
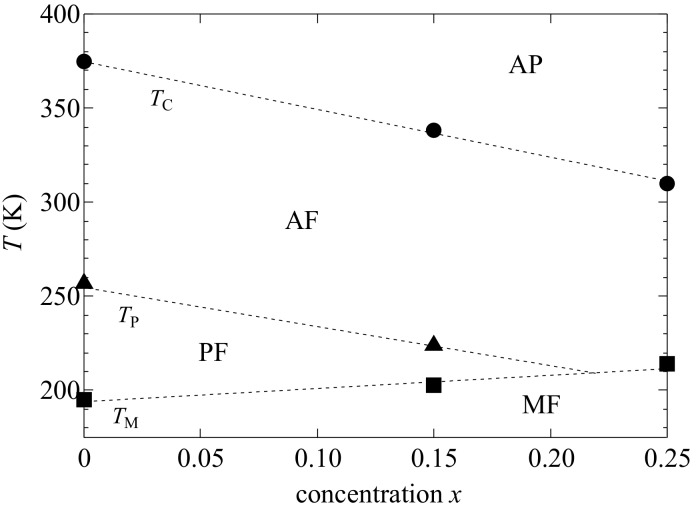
Phase diagram for Ni_2_Mn_1−*x*_Cr*_x_*Ga (0 ≤ *x* ≤ 0.25): martensitic ferromagnetic (MF) phase, premartensitic ferromagnetic (PF) phase, austenitic ferromagnetic (AF) phase, and austenitic paramagnetic (AP) phase. Data from Ref. [27]. T. Sakon, et al. *Metals* **2017**, *7*, 410. MDPI, doi:10.3390/met7100410.

**Figure 3:**
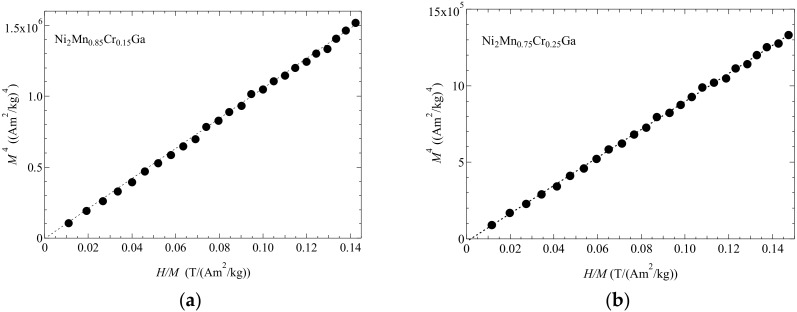
The *H*/*M* dependence of *M*^4^ for (**a**) *x* = 0.15 and (**b**) *x* = 0.25 at *T*c. The dotted straight line is the fitted line.

The crystal structure of the austenitic phase is L2_1_ cubic. Therefore, it is accurate to express the crystal structure of the premartensitic phase as a 6M structure. Figure 2 shows the structural and magnetic phase diagram of Ni_2_Mn_1−*x*_Cr*_x_*Ga for 0  ≤  *x*  ≤  0.25, where the transition temperatures were obtained from the permeability results. We measured the magnetization for *x* = 0.15 and 0.25 at *T*_C_. In our results of Ni_2_Mn_1−x_Cr*_x_*Ga, the martensitic transition temperature *T*_M_ increases with decreasing the valence electron concentration per atom, *e*/*a*. This property was also investigated by Khan et al. [29]. Concerning of Ni_2+*x*_Mn_1−*x*_Ga, the increase of *T*_M_ with increasing Ni concentration was attributed to the increase of valence electron concentration, *e*/*a* [10]. On the contrary, this tendency is not applicable to *T*_M_ of Ni_2_Mn_1−*x*_Cr*_x_*Ga. Khan et al. mentioned that, other factors like hybridization and electronegativity should be incorporated as well. This problem is open question and further study is needed. Figure 3 ((a) for *x* = 0.15 and (b) for *x* = 0.25) shows the plots of *M*^4^ versus *H*/*M*. A good linearity can be seen at the origin at *T*_C_. The results agree with the Takahashi spin fluctuation theory [12]. As well as in the case of Ni_2+*x*_MnGa_1−*x*_, the Takahashi theory has also been shown to be applicable to Ni_2_Mn_1−*x*_Cr*_x_*Ga [17]. The spin fluctuation parameter in *k*-space, *T*_A_, has been obtained from the magnetization process at *T*_C_ using the Takahashi theory [12], where the *T*_A_ values were 538 K (*x* = 0.15) and 532 K (*x* = 0.25). For the Ni_2+*x*_MnGa_1−x_ alloys, the obtained *T*_A_ values were 563 K, 566 K, and 567 K for *x* = 0.00, *x* = 0.02, and *x* =0.04, respectively [17]. These values were approximately the same as those for Ni_2_Mn_1−*x*_Cr*_x_*Ga.

### 3.2. Correlation between Magnetization and Forced Magnetostriction

**Figure 4:**
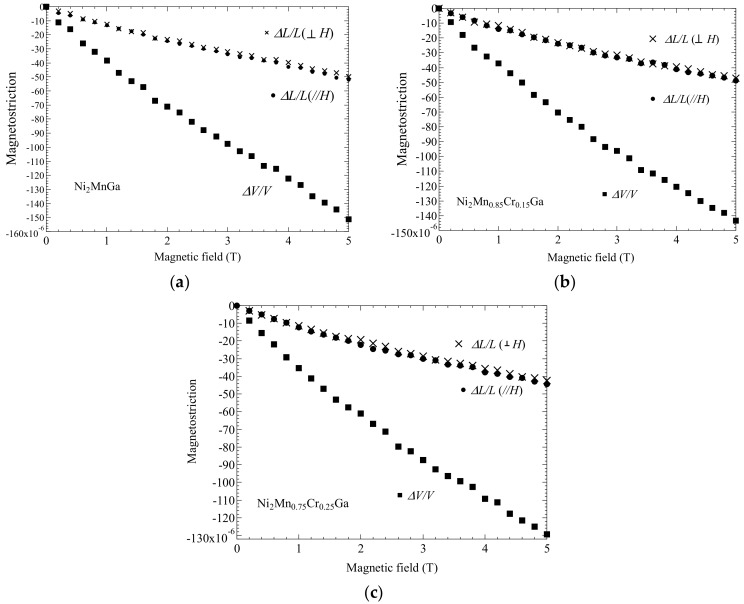
Magnetic field dependence of magnetostriction for (**a**) *x* = 0.00, (**b**) *x* = 0.15, and (**c**) *x* = 0.25 at *T*_C_. Data of (*ΔL*/*L*)_//_ in (**a**) were obtained from Ref. [16]. T. Sakon et al. *J. Appl. Phys.* **2018**, *123*, 213902, with the permission of AIP Publishing. doi:10.1063/1.5036558. Title: “Forced magnetostriction of ferromagnetic Heusler alloy Ni_2_MnGa at the Curie temperature”. Available on line: https://aip.scitation.org/doi/10.1063/1.5036558. (accessed on 6 Nov. 2019).

In this subsection, we give the measured forced magnetostrictions for Ni_2_Mn_1−*x*_Cr*_x_*Ga (for *x* = 0.00, 0.15, and 0.25) and the correlation between forced volume magnetostriction and magnetization is discussed. In order to consider the correlation between magnetization and forced magnetostriction, we evaluated the magnetostriction process in the magnetic fields. Figure 4 shows the external magnetic field dependence of the forced magnetostriction for (a) *x* = 0.00, (b) *x* = 0.15, and (c) *x* = 0.25. The forced volume magnetostriction *ΔV*/*V* was derived using Equation (2). The results shown in Figure 4 suggest that *ΔV*/*V* was approximately equal to three times (*ΔL*/*L*)_//_. V.I. Nizhankovskiia et al. and M. Matsunaga et al. have also shown that *ΔV*/*V* was equal to three times (*ΔL*/*L*)_//_ [24,26].

**Figure 5:**
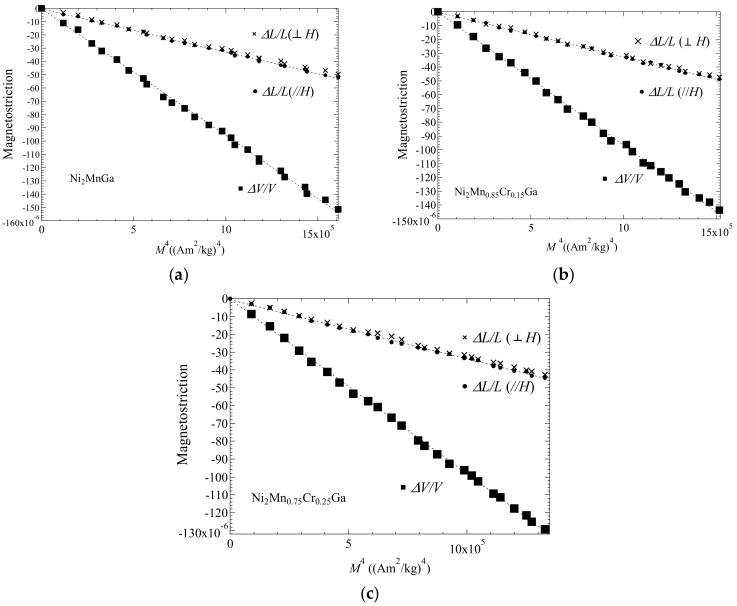
Forced magnetostriction for (**a**) *x* = 0.00, (**b**) *x* = 0.15, and (**c**) *x* = 0.25 at *T*c. The dotted straight line is the fitting line. The plots of (*ΔL*/*L*)_//_ and *M*^4^ in (**a**) were obtained from Ref. [16]. T. Sakon et al. *J. Appl. Phys.* **2018**, *123*, 213902, with the permission of AIP Publishing. doi:10.1063/1.5036558. Title: “Forced magnetostriction of ferromagnetic Heusler alloy Ni_2_MnGa at the Curie temperature”. Available on line: https://aip.scitation.org/doi/10.1063/1.5036558 (accessed on 6 November 2019).

In Figure 5a, the magnetostriction (*ΔL*/*L*)_//_, (*ΔL*/*L*)_⊥_, and *ΔV*/*V* versus *M*^4^ at *T_C_* for *x* = 0.00 are shown. Furthermore, (*ΔL*/*L*)_//_, (*ΔL*/*L*)_⊥_, and *ΔV*/*V* versus *M*^4^ at *T*_C_ for *x* = 0.15 and 0.25 are shown in Figure 5b,c, respectively. The dotted line denotes the fitting line. These plots show good linearity through the origin point at *T*_C_. The magnetostriction can be seen to be proportional to *M*^4^.

Our experimental magnetostriction results agreed with the Takahashi spin fluctuation theory [12]. The maximum value of magnetostriction at the premartensitic phase and the premartensitic transition temperature *T*_P_ indicate a linear relationship with the valence electron concentration per atom *e*/*a* [27]. Therefore, the results show that there was a correlation between the electron energy, magnetostriction, and *T*_p_. Tsuchiya et al. [31] and Matsui et al. [10,32] also showed that *T*_p_ and the martensitic transition temperature *T*_M_ are associated with *e*/*a*. Furthermore, the forced magnetostriction can also be correlated with *e*/*a*. Therefore, we investigated the correlation between *e*/*a* and the forced magnetostriction at *T*_C_.

**Figure 6:**
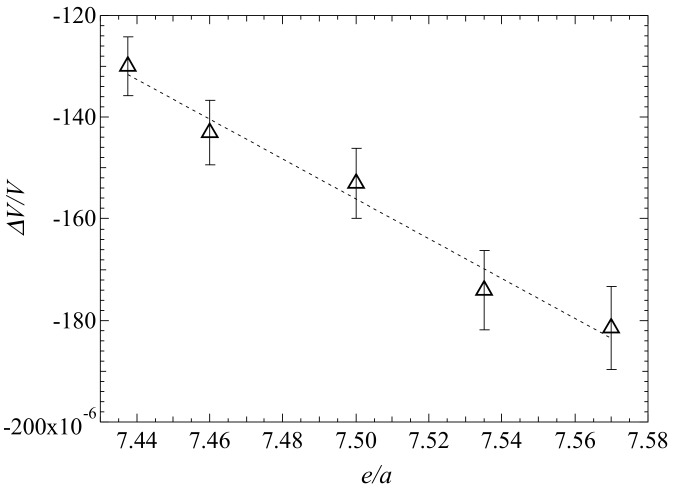
Forced volume magnetostriction *ΔV*/*V* versus *e*/*a* at 5 T. The plots with *e*/*a* = 7.535 (Ni_2.02_MnGa_0.98_) and *e*/*a* = 7.570 (Ni_2.04_MnGa_0.96_) were obtained from Ref. [17] T. Sakon et al. *Materials* **2018**, *11*, 2115. MDPI, doi:10.3390/11112115. The dotted straight line is the fitting line.

Figure 6 shows the forced volume magnetostriction *ΔV*/*V* versus *e*/*a* at *T*_C_ under 5 T for Ni_2_Mn_1−*x*_Cr*_x_*Ga and Ni_2+*x*_MnGa_1−*x*_ [17]. The values of *ΔV*/*V* calculated using the experimental results of (*ΔL*/*L*)_//_ for Ni_2+*x*_MnGa_1−*x*_ (*x* = 0.02 and 0.04) are also shown [17]. Error bars are indicated for each point. It can be seen that *ΔV*/*V* was approximately proportional to *e*/*a*. Thus, the forced volume magnetostriction reflects the electronic state of the alloys. In a previous investigation, we studied magnetostriction near the premartensitic phase and showed a correlation between the electron energy, magnetostriction, and *T*_p_ [27]. Uba et al. carried out theoretical band calculations for Ni_2_MnGa [33]. The spin-polarized partial densities of states (DOS) of Ni_2_MnGa for the austenitic parent phase (L2_1_) structure, obtained from relativistic generalized gradient approximation calculations (GGA), were shown. The GGA results agreed well with previous band structure calculations, as shown in Ref. [33]. In the austenite phase, the Mn 3d states (*e*_g_ and *t*_2g_) and Ni 3d states (*e* and *t*_2g_) were located at the Fermi level. The obtained GGA energy band structure indicated that five energy bands, from 29 to 33, crossed the Fermi level. The value of the magnetization increased when the external magnetic fields were increased, due to the itinerant magnetism. The magnetization and magnetostriction were induced by magnetic fields. Therefore, it has been concluded that the forced magnetostriction is associated with the band energy. Thus, the magnetostriction is associated with the magnetization.

### 3.3. Comparison between the Forced Magnetostriction in the Premartensitic Phase and that at T_C_

**Figure 7:**
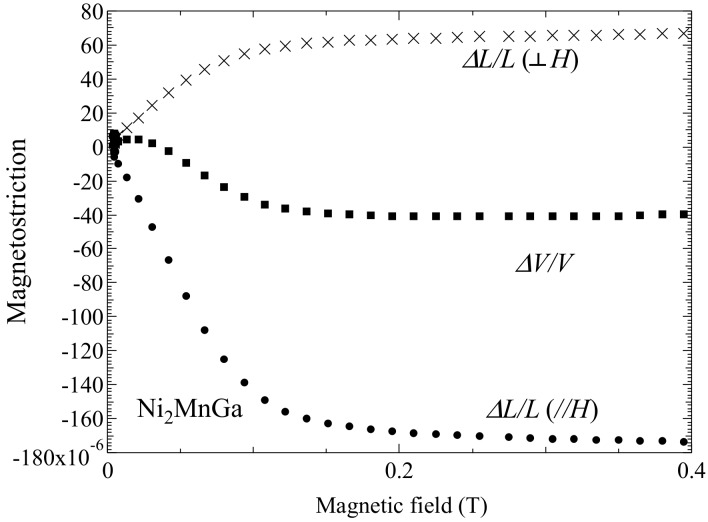
Magnetic field dependence of the magnetostriction at *T* = 251 K in the premartensitic phase.

**Figure 8:**
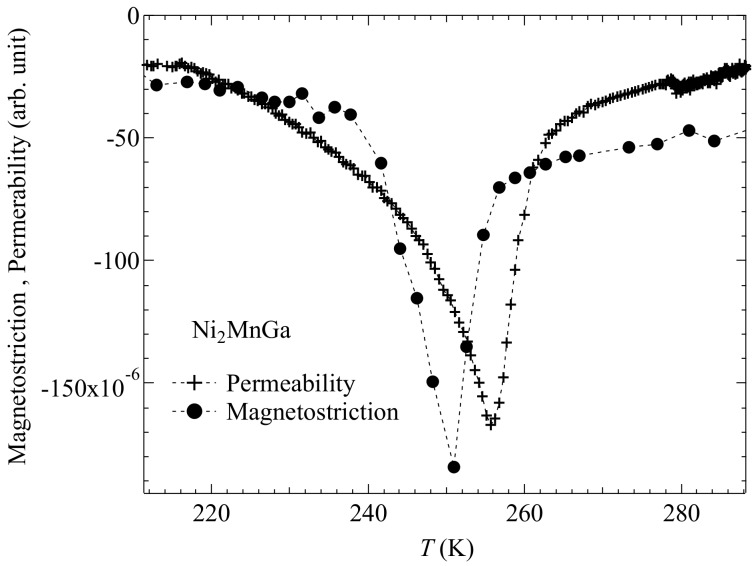
Temperature dependence of permeability at zero magnetic fields and magnetostriction of Ni**_2_**MnGa at 1.6 T. Dotted lines are guides to eyes. The plots were obtained from Ref. [27]. T. Sakon et al. *Metals* **2017**, *7*, 410. MDPI, doi:10.3390/met7100410.

The magnetostriction at the premartensitic phase was compared to that at *T*_C_. Figure 7 shows the magnetostriction for *x* = 0.00 (Ni_2_MnGa) in the premartensitic phase at 250 K. The forced magnetostriction perpendicular to the magnetic field, ${\left(\Delta L/L\right)}_{⊥}$ was smaller than that of the longitudinal magnetostriction parallel to the magnetic field, (*ΔL*/*L*)_//_. Moreover, the magnetostriction varied in a low magnetic field, below 0.2 T. These properties contradicted the forced magnetostriction at *T*_C_, as shown in Figure 4a. At *T*_C_, (*ΔL*/*L*)_//_ was approximately the same value as ${\left(\Delta L/L\right)}_{⊥}$ and the forced magnetostriction gradually decreased with an increasing magnetic field. Salazar Mejía et al. performed resonant ultrasound spectroscopy measurements of Ni_2_MnGa [34]; when cooling from high temperatures, a sharp decrease in the ultrasound frequency *f*(*T*) was observed at *T*_P_ and, for the magnetic susceptibility, a decrease (which corresponded to the premartensitic transition) was observed at *T*_P_ [34]. Further, *f*(*T*) showed no thermal hysteresis near *T*_P_. The permeability results also indicated a decrease at *T*_P_, and the permeability did not show thermal hysteresis near *T*_P_ [27]. Therefore, the premartensitic transition was a second-order transition. The shear elastic coefficient *C*’ (*C*’ = (*C*_11_ − *C*_12_)/2) decreased near *T*_P_ [35]. From the ultrasound experiments, lattice softening occurred near *T*_P_. The premartensitic transition between the austenitic phase and premartensitic phase originated from softening of the shear elastic coefficient *C*’ [35] corresponding to a minimum of the slowest transversal phonon branch [3,36,37,38]. Near *T*_P_, a large magnetostriction was induced with a weak magnetic field (0.2 T), owing to lattice softening. Conversely, *f*(*T*) did not indicate a clear decrease or anomaly near *T*_C_, and lattice softening was negligible at *T*_C_. Thus, the forced magnetostriction decreased gradually with an increasing magnetic field at *T*_C_. Figure 8 shows plots of the temperature dependence of permeability and magnetostriction for Ni_2_MnGa. The permeability presents a clear dip around the temperature of the premartensitic transition (*T*_P_ = 255 K). The absolute value of the magnetostriction indicates a maximum value at 251 K, which is just below *T*_P_. The peak temperature of the magnetostriction (251 K) is 5 K lower than the peak temperature of the permeability (256 K). Mañosa et al. performed ultrasound spectroscopy and permeability measurements [35]. The temperature dependence of the shear elastic coefficient *C*’ and the permeability presented a clear dip around *T*_P_. The peak temperature of *C*’ (228 K) was 5 K lower than the peak temperature of the permeability (233 K). These results indicate that the peak temperature of both the magnetostriction and *C*’ were 5 K lower than that of the permeability. In this regard, it is conceivable that the magnetostriction is correlated with the lattice softening.

Zelený et al. performed a theoretical study on the phase transition from the cubic austenitic Heusler structure to low-symmetry martensitic structures [39]. They used ab initio calculations combined with the generalized solid state nudged elastic band method (G-SSNEB) to determine the minimum energy pathway in the crystal lattice. For alloys close to stoichiometric alloys, the modulated 6M premartensitic structure appeared just above the temperature of martensitic transition as a precursor to the martensitic transition [40]. This result was the same as that found by the X-ray measurements performed by Singh et al. [30].

In order to classify with different lattices, a referential co-ordinate, which is independent of the characteristic lattice geometry or arrangement of atoms along the transition pathway, has been introduced [39]. The reaction co-ordinate, *RC*, defines a referential co-ordinate which universally defines the advance of transition between austenite (*RC* = 0) and fully transformed martensite (*RC* = 1), or the transition between 6 M premartensite (*RC’* = 0) and fully transformed martensite (*RC’* = 1). All lattice energies are fully relieved relative to the transition pathway, including both unit-cell and atomic variance. The numerically computed minimum energy pathways of Ni_2_MnGa along *RC* and *RC’* can be seen in Figure 2a,b in Ref. [39]. As for the austenite–martensite transition, *RC* is applied as the parameter of the phase transition. In this case, the phase transitions from the austenitic phase to the 10 M or 14M martensite phase directly. It has been insisted that the transition proceeds as A→6M→10M (RC = 0.29, RC’ = 0), which differs with the experimental X-ray study of Ni_2_MnGa [30]. Zelenyý et al. mentioned that, for the A→14M transition, there is an energy barrier of a certain degree in the transition at *RC* ≈ 0.15 [39]. Thus, it can be concluded that the A→6M→10M transition is easier process than the A→14M transition. Additional bridging between the experimental and theoretical investigations for the crystal structure in the ground state of the martensite phase is needed.

Stoichiometric Ni_2_MnGa presents the premartensitic transition A→6M. This transition is led by a gradual softening of the *C′* in the cooling process. The *C′* value is linked with the TA_2_ [ξξ0] phonon branch. Therefore, it has been concluded that the transition will be realized by a tetragonal lattice distortion [36]. The calculated A→6M pathway involves a “small” tetragonal distortion of the austenitic lattice. The experimental results on the temperature dependence of the permeability obtained in our study and in other studies indicate that the premartensite transition is a second-order transition. The theoretical calculation results by Zelenyý et al. indicated a gradual transition from the austenite phase to the 6M martensite phase. Their results also support a second-order transition.

As previously mentioned, the crystal structure of Ni_2_MnGa in the premartensitic phase is a 6M incommensurate structure [30]. In the premartensitic phase, the crystal symmetry became lower than that of the cubic L2_1_ structure in the austenitic phase. Therefore, the magnetism and crystallographic structure should be further evaluated.

### 3.4. Consideration of the Origin of the Large MFIS at the Martensitic Phase and Magnetostriction at the Premartensitic Phase

In this subsection, we consider the physical origin of the large magnetostrictions of Ni_2_MnGa. It has been proposed that the band structure of Ni_2_MnGa is related to the magnetostriction. Ayuela et al. performed a band calculation study and investigated the correlation between the *c*/*a* ratio and the difference of the total energy, *ΔE*, between the L2_1_ cubic and tetragonal structures due to the tetragonal distortion [41]. For *c*/*a* < 1, there was a shallow hollow and the minimum point of the energy was at *c*/*a* = 0.95; this point corresponds to the 10 M martensitic structure. The depth of the hollow (potential barrier) of *ΔE* was only 0.02 mRy = 0.26 meV = 3.4 T. Therefore, a large MFIS occurred in the magnetic fields at the martensitic phase. Tsuchiya et al. mentioned that the 14 M structure is realized for *c*/*a* = 0.89 [31]. The band structure changes with the temperature and, so, a 10 M or 14 M structure is realized.

**Figure 9:**
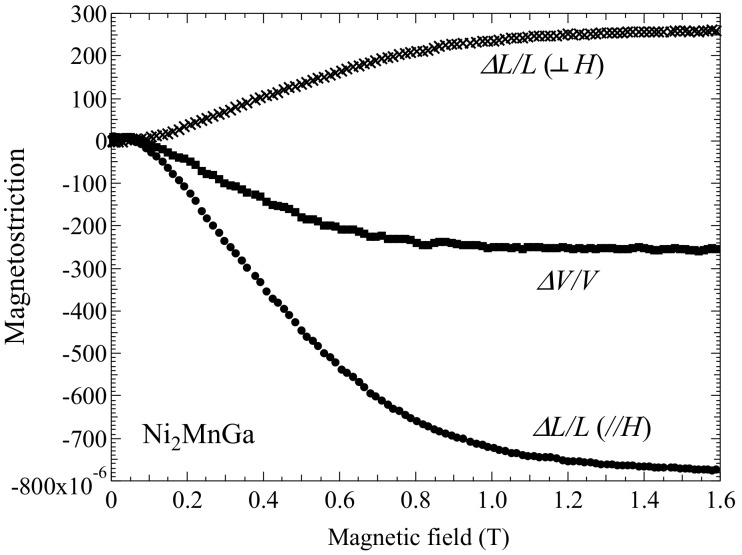
Magnetic field dependence of the magnetostriction at *T* = 185 K in the martensitic phase.

Figure 9 shows the magnetic field dependence of the magnetostriction at 185 K in the martensitic phase. The shape of the magnetostriction in the martensite phase (Figure 9) and that in the premartensitic phase (Figure 7) resemble the shape of the magnetostriction for Ni [42]. For Ni_2_MnGa, band calculations have indicated that the Mn 3d up (majority) spin peaks are located at around 1 and 3 eV below *E*_F_. On the contrary, the Mn down (minority) spin peak is located at around 1.5 eV above *E*_F_ [40]. The Mn DOS around *E*_F_ is much smaller than that at the peaks. On the contrary, Ni has a large down spin DOS around *E*_F_. Matsui et al. mentioned the difference between Co_2_MnGa and Ni_2_MnGa [10]. Co_2_MnGa is a L2_1_-type half-metallic Heusler alloy, with a spin polarization ratio of 48% [43]. The Fermi level falls within the gap or the pseudo-gap, and an almost perfect spin-polarization at the Fermi level is preserved [44]. Therefore, even if the *e*/*a* ratio is varied, the L2_1_ crystal structure is stable. Thus, the martensitic transition does not occur at low temperatures. As for Ni_2_MnGa, the peak of the Ni down spin is located around 10 mRy = 0.13 eV and there is a large down spin DOS around *E*_F_ [10,40]. Consequently, the DOS around the *E*_F_ is sensitive to both the temperature and the *e*/*a* value. Matsui et al. mentioned that the martensitic transition is sensitive to *e*/*a* and that the A→10M and A→14M transitions occur for *e*/*a* > 7.65 and *e*/*a* > 7.70, respectively [10]. Furthermore, experimental results have shown that the premartensitic phase appears for *e*/*a* < 7.65 [27]. This is due to the shallow hollow of *ΔE*. As a result, softening of the lattice occurs and a relatively large magnetostriction is caused by the magnetic field.

In favor of the applied use of the large magnetostriction of Ni_2_MnGa-type Heusler alloys, many investigations have been performed, and large MFIS alloys have been found [1,6,45,46]. Magnetic actuators and tremblers have been made, which are commercially available [47]. These materials are single crystals. Magnetic actuation by means of Ni_2_MnGa-type single crystal alloys can generate large displacement (of a few percent), due to the large MFISs of these alloys. On the other hand, the magnetostriction values of the polycrystal alloys are approximately two columns smaller than those of the single crystals. However, in general, polycrystal crystals are superior to single crystals, in terms of machine properties such as ductility or toughness [48]. There are some possibilities for their use in magnetic sensors by means of the magnetostriction of polycrystals. In the martensitic phase, the value of the magnetostriction is larger than that in the austenitic phase. Therefore, for commercial use, it is valuable to search for alloys which have *T*_M_ values higher than that of room temperature. For Ni_2+x*X*_Mn_1__−_*_x_*Ga (0.15 < *x* < 0.20), the ferromagnetic martensitic (FM) phase has been realized at room temperature [49]. For Ni_50+*x*_Mn_12.5_Fe_12.5_Ga_25−*x*_ (3 < *x* < 5), the FM phase has also been realized at room temperature [50]. These polycrystal alloys are thus candidates for use as magnetic functional materials. Further investigation is needed for the development of applied use of such materials.

## 4. Conclusions

The correlation between forced magnetostriction and magnetization was evaluated using the SCR spin fluctuation theory of an itinerant ferromagnet for the ferromagnetic Ni_2_Mn_1−*x*_Cr*_x_*Ga-type Heusler alloys. The magnetization results at *T*_C_ suggest that the magnetic field is directly proportional to *M*^5^, which agreed with the Takahashi spin fluctuation theory. Thus, the forced longitudinal magnetostriction *ΔL*/*L* and forced volume magnetostriction *ΔV*/*V* at *T*_C_ are proportional to *M*^4^; furthermore, the plots crossed the origin point. This result is in good agreement with the Takahashi spin fluctuation theory. An experimental study was carried out and the results of the measurement agreed with the theory. The value of forced magnetostriction was proportional to the valence electron concentration per atom (*e*/*a*). Therefore, the forced magnetostriction reflects the electronic state of the alloys.

In addition, we evaluated the difference between the magnetostriction near *T*_P_ and that at *T*_C_. The magnetostriction near *T*_P_ caused lattice softening. Conversely, lattice softening was negligible at *T*_C_. The magnetostriction at *T*_C_ was due to the itinerant electron magnetism. The DOS around the *E*_F_ was sensitive to the temperature and the *e*/*a*, and the martensitic transition was sensitive to *e*/*a*. The A→10M and A→14M transitions occur for *e*/*a* > 7.65 and *e*/*a* > 7.70, respectively. The experimental results indicate that the premartensitic phase appears for *e*/*a* < 7.60. This is due to the shallow hollow of *ΔE*. As a result, softening of the lattice occurs, and a relatively large magnetostriction is caused by the magnetic field.
